# Supplementary material for: Allosteric inhibition of HTRA1 activity by a conformational lock mechanism to treat age-related macular degeneration
Source: Nat Commun. 2022 Sep 5;13:5222. doi: 10.1038/s41467-022-32760-9 (PMC9445180; doi:10.1038/s41467-022-32760-9)
Supplement: Supplementary file 3 — Reporting Summary [file 41467_2022_32760_MOESM3_ESM.pdf]

nature portfolio

Corresponding author(s): Daniel Kirchhofer, PhD

Last updated by author(s): Jun 8, 2022

Reporting Summary

Nature Portfolio wishes to improve the reproducibility of the work that we publish. This form provides structure for consistency and transparency in reporting. For further information on Nature Portfolio policies, see our Editorial Policies and the Editorial Policy Checklist.

Statistics

For all statistical analyses, confirm that the following items are present in the figure legend, table legend, main text, or Methods section.

n/a	Confirmed	
☐	☒	The exact sample size (*n*) for each experimental group/condition, given as a discrete number and unit of measurement
☐	☒	A statement on whether measurements were taken from distinct samples or whether the same sample was measured repeatedly
☒	☐	The statistical test(s) used AND whether they are one- or two-sided<br>*Only common tests should be described solely by name; describe more complex techniques in the Methods section.*
☒	☐	A description of all covariates tested
☒	☐	A description of any assumptions or corrections, such as tests of normality and adjustment for multiple comparisons
☐	☒	A full description of the statistical parameters including central tendency (e.g. means) or other basic estimates (e.g. regression coefficient) AND variation (e.g. standard deviation) or associated estimates of uncertainty (e.g. confidence intervals)
☒	☐	For null hypothesis testing, the test statistic (e.g. *F*, *t*, *r*) with confidence intervals, effect sizes, degrees of freedom and *P* value noted<br>*Give P values as exact values whenever suitable.*
☒	☐	For Bayesian analysis, information on the choice of priors and Markov chain Monte Carlo settings
☒	☐	For hierarchical and complex designs, identification of the appropriate level for tests and full reporting of outcomes
☒	☐	Estimates of effect sizes (e.g. Cohen's *d*, Pearson's *r*), indicating how they were calculated

Our web collection on statistics for biologists contains articles on many of the points above.

Software and code

Policy information about availability of computer code

Data collection	For Xray data collection, refinement and graphics we used the following software versions: PHENIX (phenix.refine: 1.9_1692)<br>PyMOL v1.8.6.2 Enhanced for Mac OS X; BUSTER 2.11.6; CCP4 7.1 dials -v1-9-2; XDS 1.1.5; Phaser 2.5.5; Coot 0.8.6; refmac 5.8.0049, Aimless Version: 1.1.7; STARANISO OpenMP version: 1.10.9<br>for cryoEM data collection, refinement and graphics we used the following software versions: SerialEM 3.7.11, cryoSPARC v3.2, cisTEM, Relion 3.0, PHENIX (phenix.refine and phenix.validation_cryo-EM) with build in MolProbity scoring software 4.5.<br>For Molecular dynamics simulation we used the following software versions: AMBER (https://ambermd.org), VMD 1.9.3 (https://www.ks.uiuc.edu/Research/vmd/), CPPTRAJ (https://ambermd.org), Bio3d (http://thegrantlab.org/bio3d/)
Data analysis	Structural data was analyzed and visualized using PyMOL 2.4.0 and UCSF ChimeraX 0.93<br>SPR data in Supplementary Table 1, 4-7 were analyzed by using the GE Biacore S200 instrument and the Biacore T200 evaluation software version 3.0, respectively.<br>Enzymatic data was analyzed with Graphpad Prism 9. SDS-PAGE, western blot or fluorescent gels were analyzed using Gel Doc Imager Software (Biorad)

For manuscripts utilizing custom algorithms or software that are central to the research but not yet described in published literature, software must be made available to editors and reviewers. We strongly encourage code deposition in a community repository (e.g. GitHub). See the Nature Portfolio guidelines for submitting code & software for further information.

Data

Policy information about availability of data

All manuscripts must include a data availability statement. This statement should provide the following information, where applicable:

- Accession codes, unique identifiers, or web links for publicly available datasets
- A description of any restrictions on data availability
- For clinical datasets or third party data, please ensure that the statement adheres to our policy

The atomic coordinates have been deposited in the Protein Data Bank (PDB) with the accession code 7SJO for the HtrA1PD/SA:Fab15H6.v4 complex, 7SJN for HtrA1PD:Fab15H6.v4, 7SJM for the apo Fab15H6.v4 and 7SIP for the Fab15H6.v4 + LoopA-HtrA1 peptide.

The EM maps have been deposited in the EMDB with the accession codes EMD-26163 for the HtrA1PD/SA:Fab15H6.v4 complex and EMD-25162 for the HtrA1PD:Fab15H6.v4. Other structures used as alignments for illustrations and illustrations are available in the PDB, including 3NZI, 3TJO and 3TJN. All reagents are available from the lead contact under a material transfer agreement with Genentech. Source data are provided with this paper.

Field-specific reporting

Please select the one below that is the best fit for your research. If you are not sure, read the appropriate sections before making your selection.

☒ Life sciences ☐ Behavioural & social sciences ☐ Ecological, evolutionary & environmental sciences

For a reference copy of the document with all sections, see nature.com/documents/nr-reporting-summary-flat.pdf

Life sciences study design

All studies must disclose on these points even when the disclosure is negative.

Sample size	All data represented in this study was done at least in triplicate, N>3 and data is represented as mean values and standard deviation. Generally, sample size was not predetermined for this study. Cryo-EM dataset sizes were determined by the need to attain target structural resolutions.
Data exclusions	No data was excluded.
Replication	SPR experiments for determining Kd values were carried out at least three times on different experimental days using freshly made reagents and newly made dilution series of analytes (Supplementary Table 1, 4-7). All attempts at replication were successful.<br><br>Enzymatic assays were carried out at least three times on different experimental days using freshly made reagents. All attempts at replication were successful.<br><br>Western blots, SDS-PAGE gels and fluorescent gels were repeated at least 3 times and representative images are shown in the paper. Appropriate controls were run in parallel. All attempts at replication were successful.
Randomization	Our experiments were not randomized. This statistical consideration is not relevant to our study because of the nature of biochemical, structural, and electrophysiological experiments performed in the work.
Blinding	The investigators were not blinded. Blinding is not technically or practically feasible for the experiments in this work.

Reporting for specific materials, systems and methods

We require information from authors about some types of materials, experimental systems and methods used in many studies. Here, indicate whether each material, system or method listed is relevant to your study. If you are not sure if a list item applies to your research, read the appropriate section before selecting a response.

Materials & experimental systems

n/a	Involved in the study	
☐	☒	Antibodies
☐	☒	Eukaryotic cell lines
☒	☐	Palaeontology and archaeology
☒	☐	Animals and other organisms
☒	☐	Human research participants
☒	☐	Clinical data
☒	☐	Dual use research of concern

Methods

n/a	Involved in the study	
☒	☐	ChIP-seq
☒	☐	Flow cytometry
☒	☐	MRI-based neuroimaging

Antibodies

Antibodies used	anti-HtrA1 Fab15H6.v4 (Genentech Inc., available upon request)<br>anti-HtrA1/HtrA2:7816 19G10 mouse IgG2a monoclonal antibody (Genentech)
Validation	Antibodies used in this studies have been validated internally and published in peer reviewed journals (e.g. Tom I. et al. 2020 PNAS)

Eukaryotic cell lines

Policy information about cell lines

Cell line source(s)	*State the source of each cell line used.*
Authentication	*Describe the authentication procedures for each cell line used OR declare that none of the cell lines used were authenticated.*
Mycoplasma contamination	*Confirm that all cell lines tested negative for mycoplasma contamination OR describe the results of the testing for mycoplasma contamination OR declare that the cell lines were not tested for mycoplasma contamination.*
Commonly misidentified lines<br>(See ICLAC register)	*Name any commonly misidentified cell lines used in the study and provide a rationale for their use.*
