# Supplementary material for: Allosteric inhibition of HTRA1 activity by a conformational lock mechanism to treat age-related macular degeneration
Source: Nat Commun. 2022 Sep 5;13:5222. doi: 10.1038/s41467-022-32760-9 (PMC9445180; doi:10.1038/s41467-022-32760-9)
Supplement: Supplementary file 1 — Supplementary Information [file 41467_2022_32760_MOESM1_ESM.pdf]

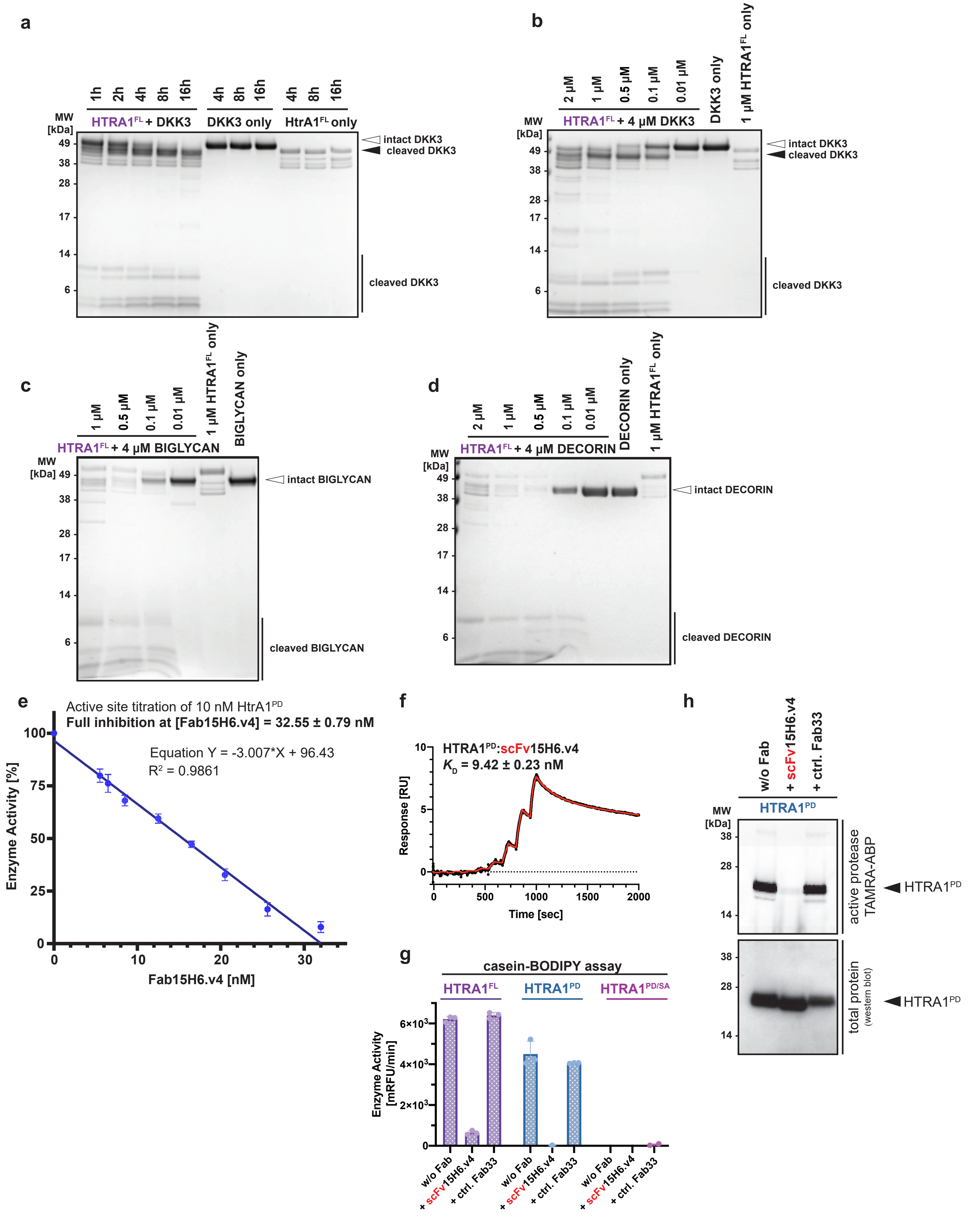

Supplementary Figure S1

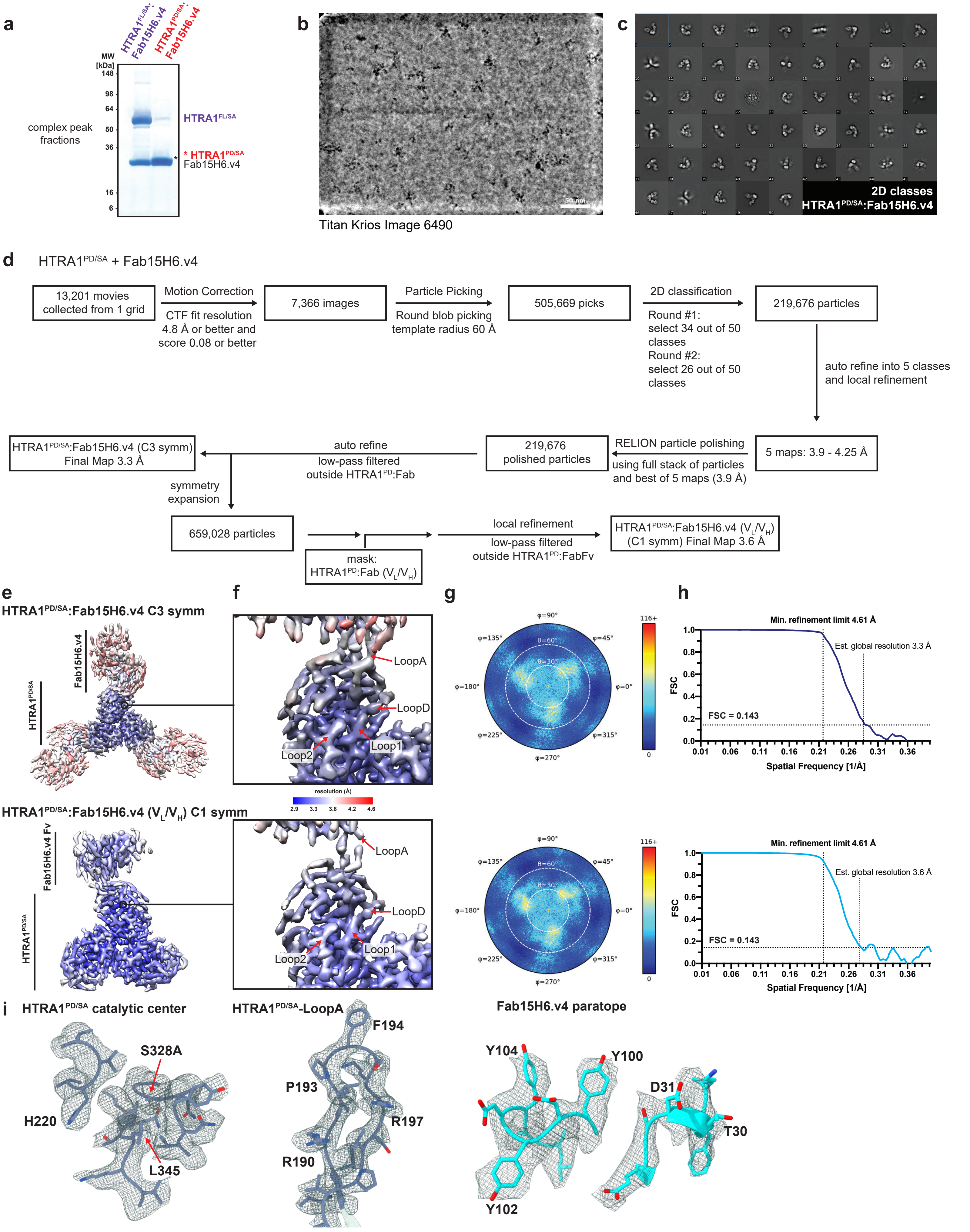

Supplementary Figure S2

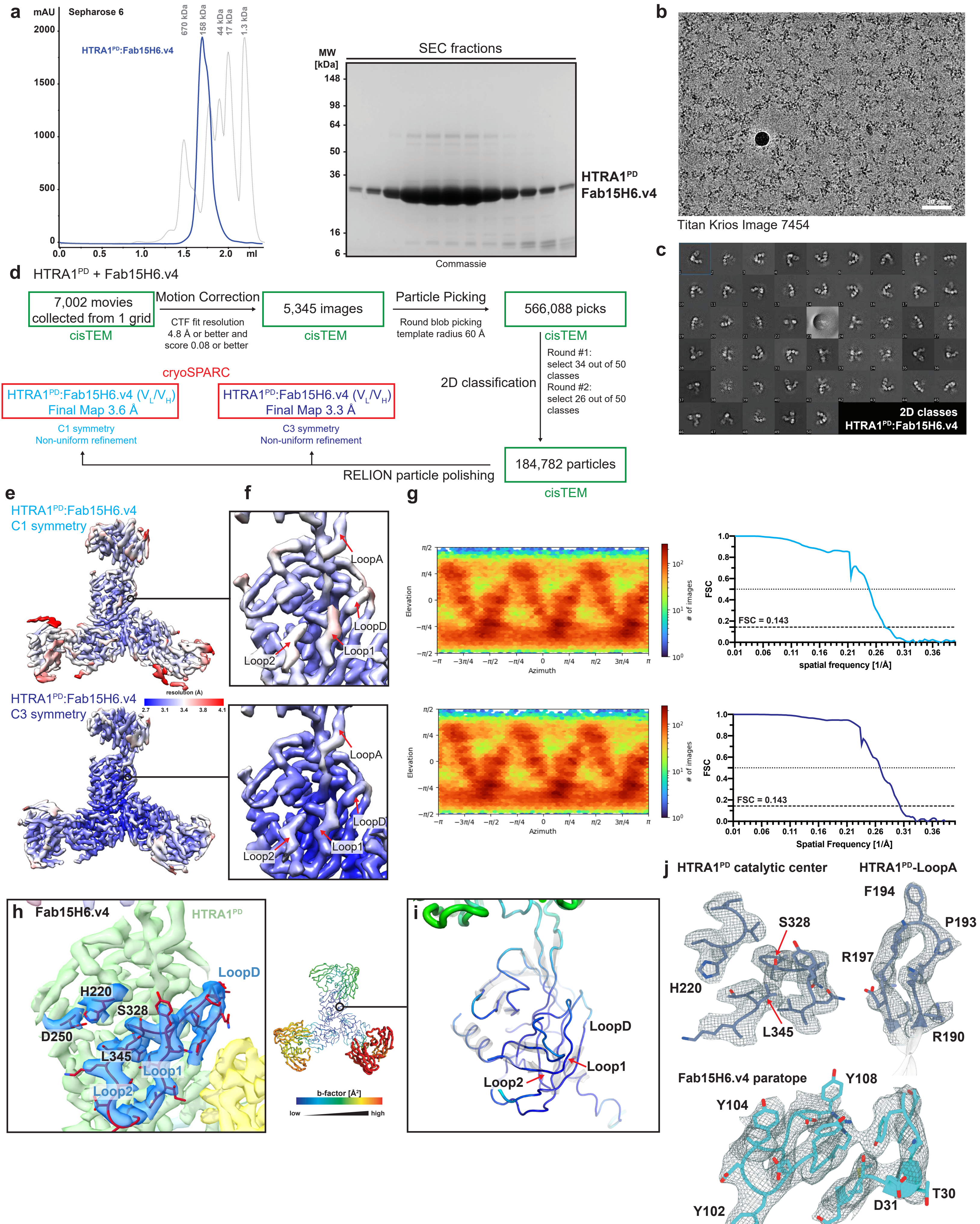

Supplementary Figure S3

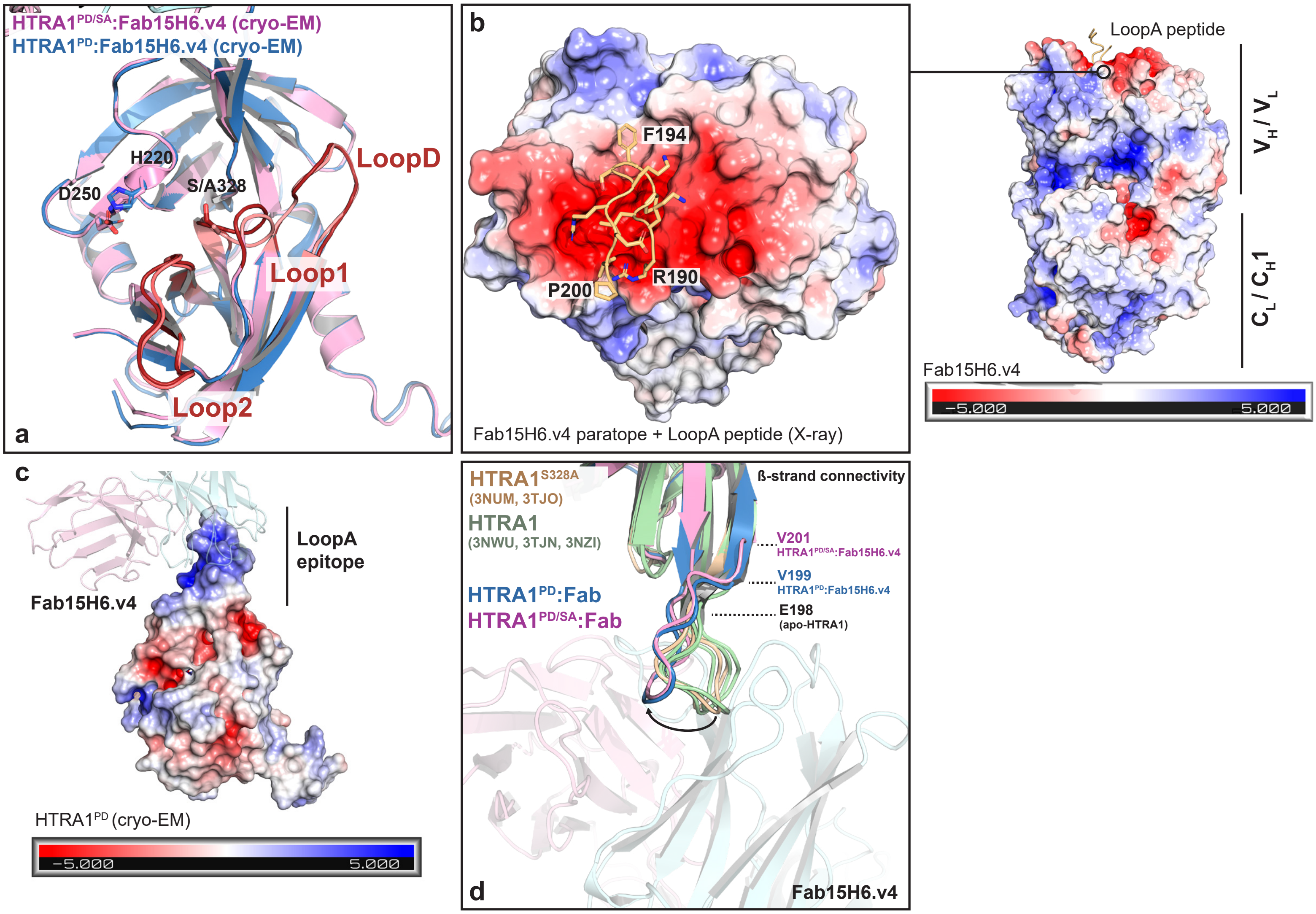

Supplementary Figure S4

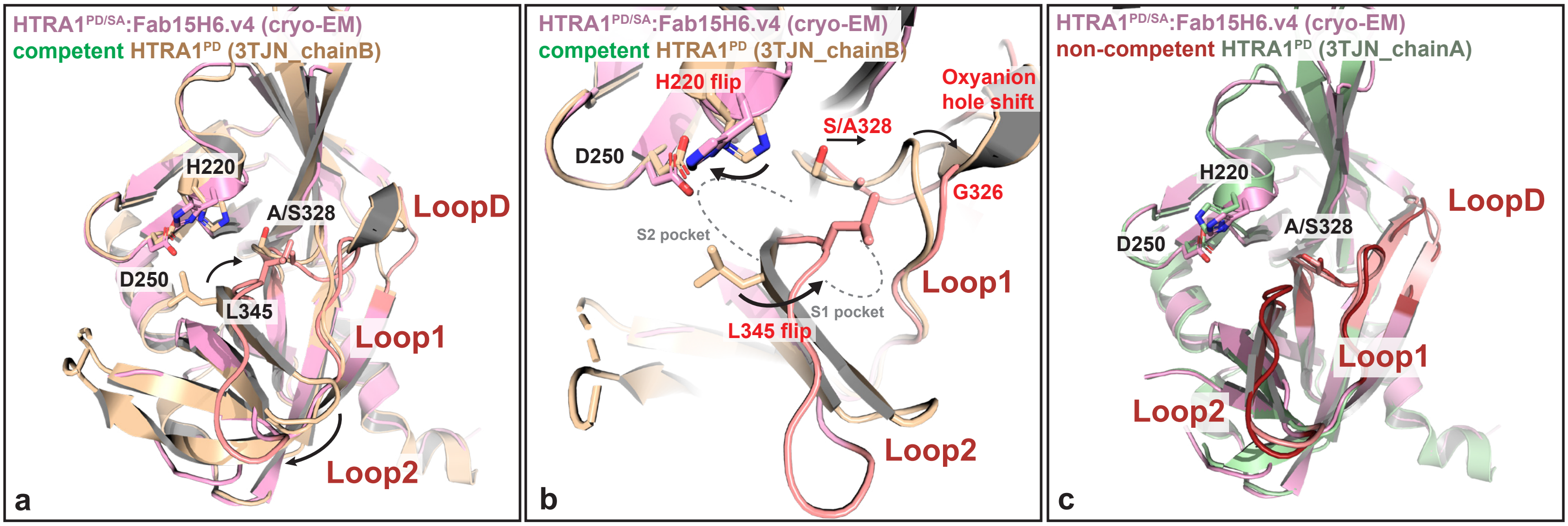

Supplementary Figure S5

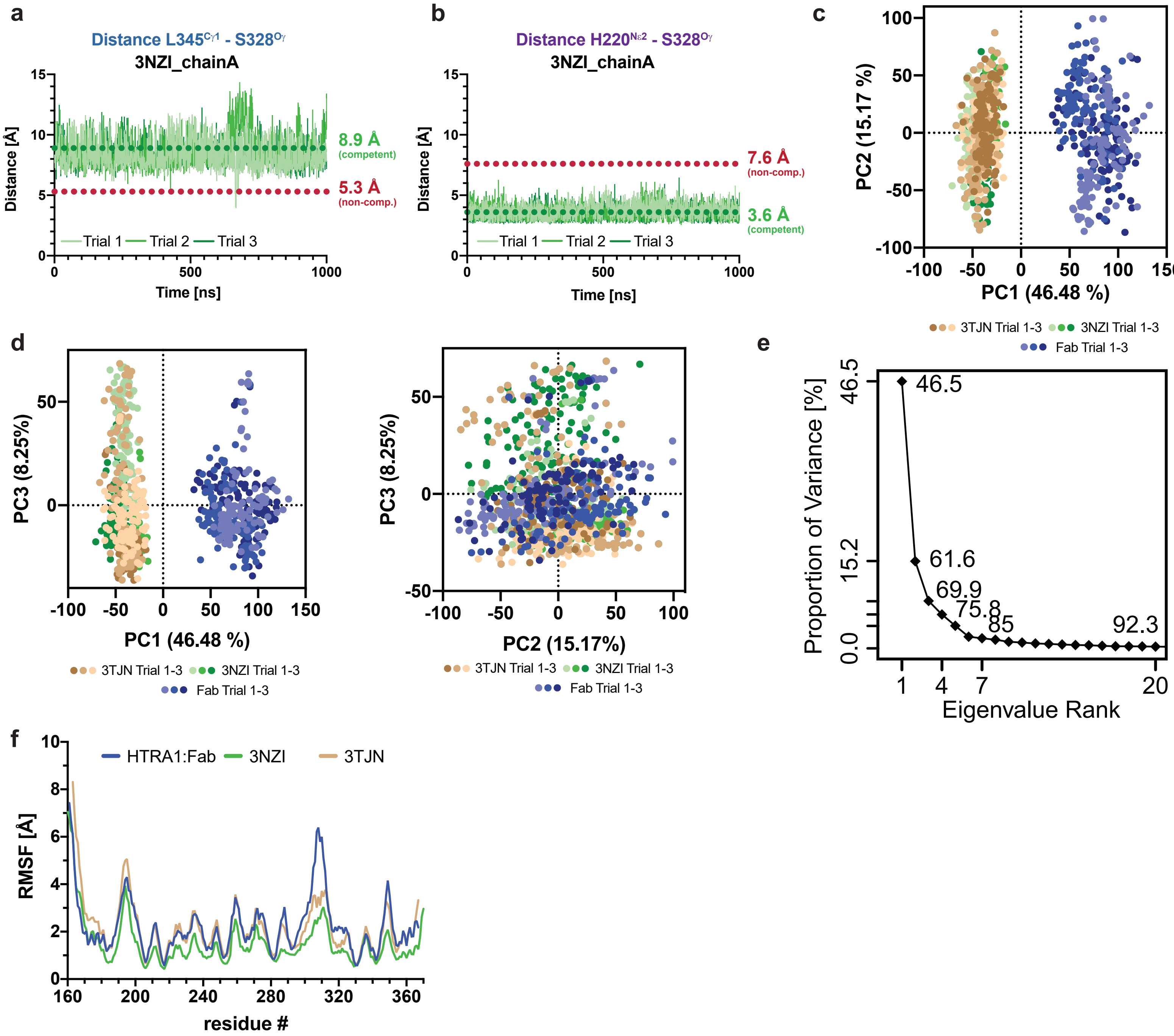

Supplementary Figure S6

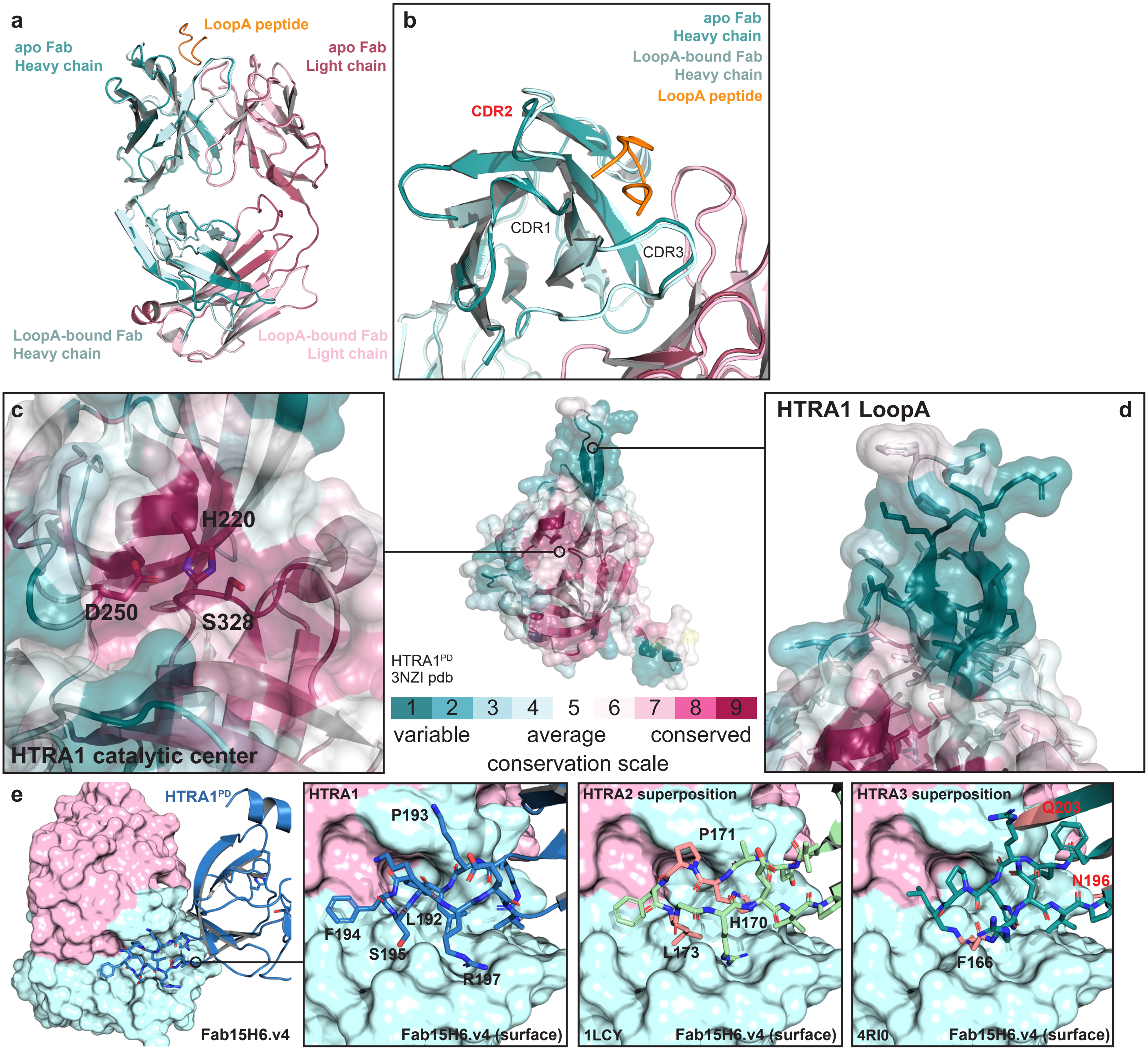

Supplementary Figure S7

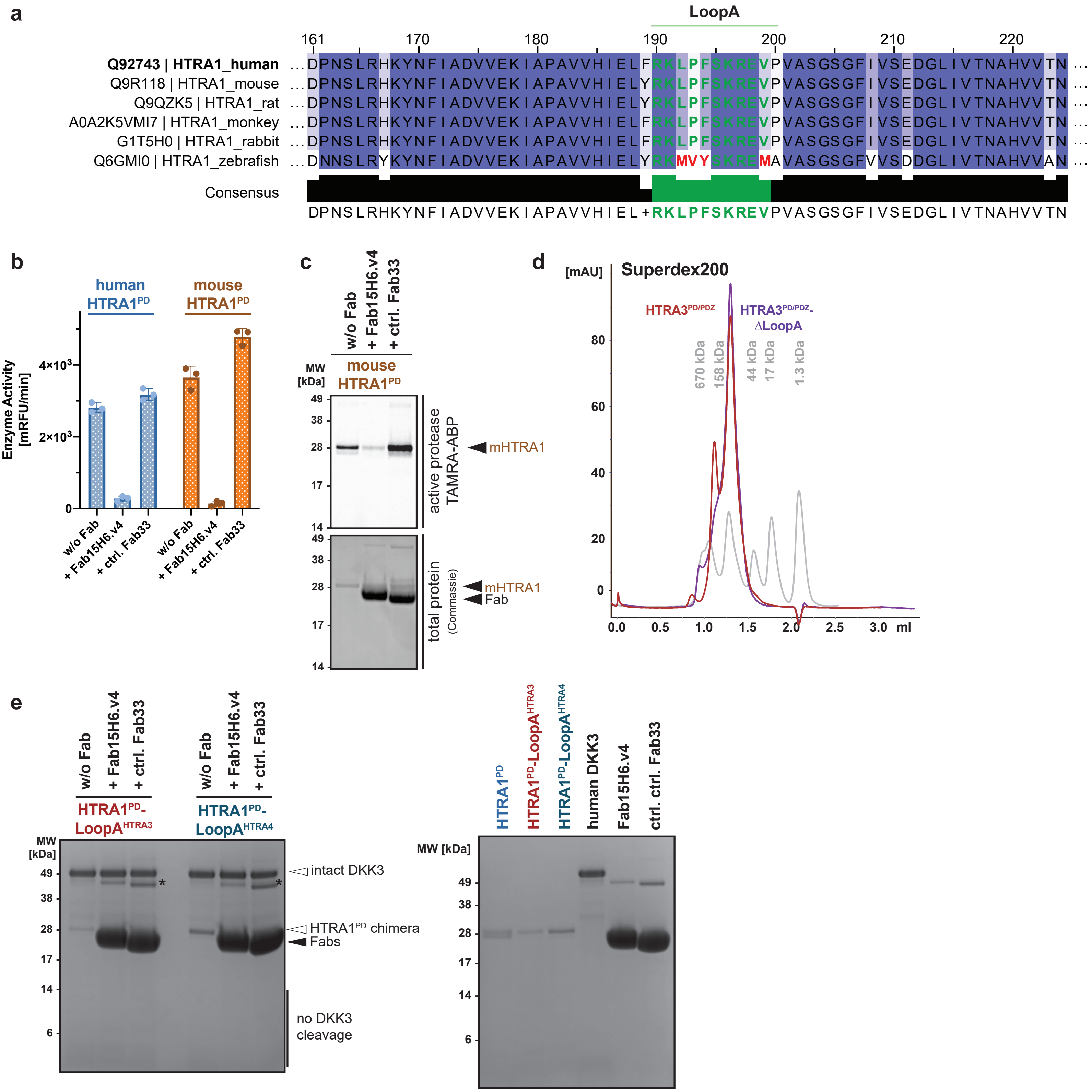

Supplementary Figure S8

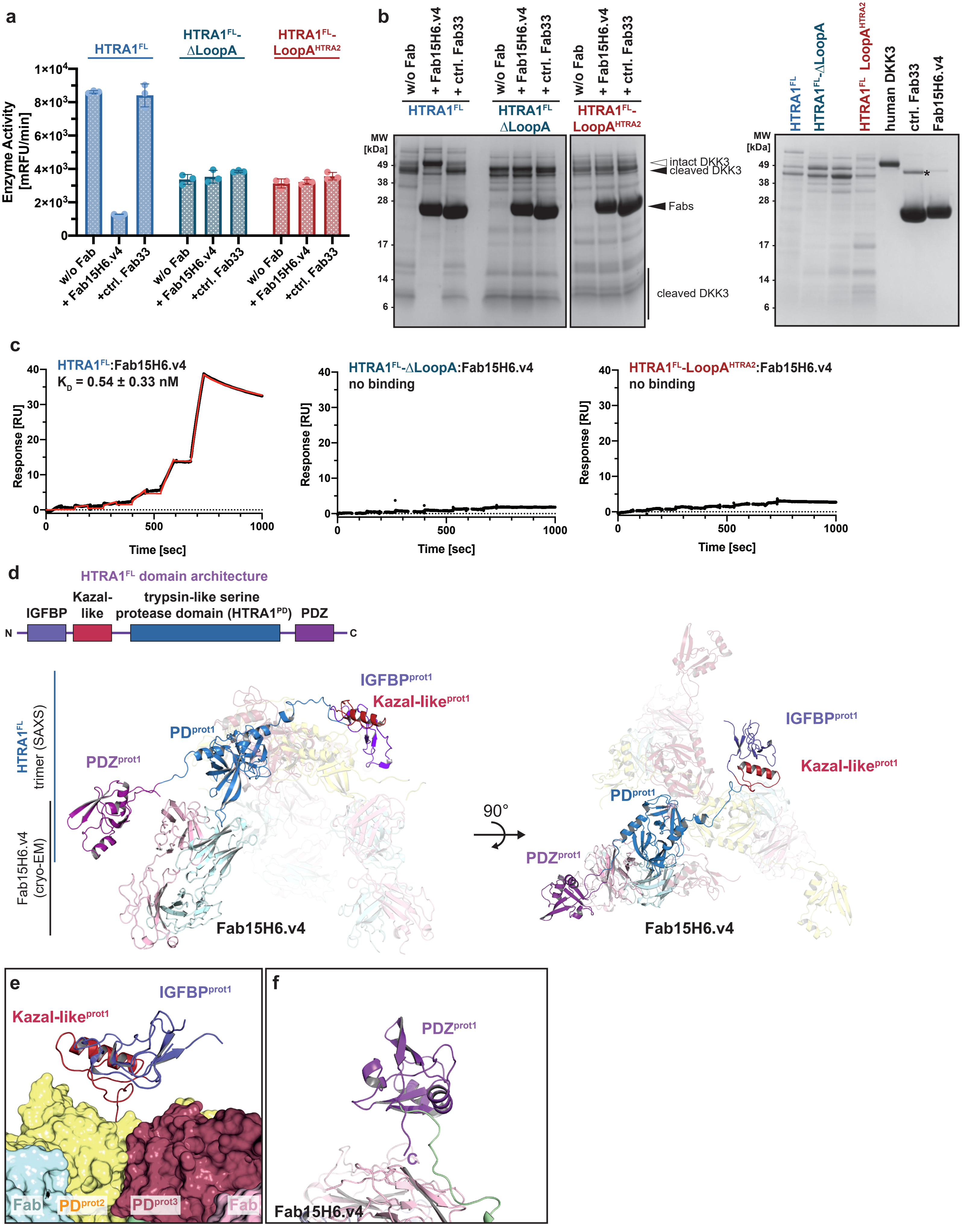

Supplementary Figure S9

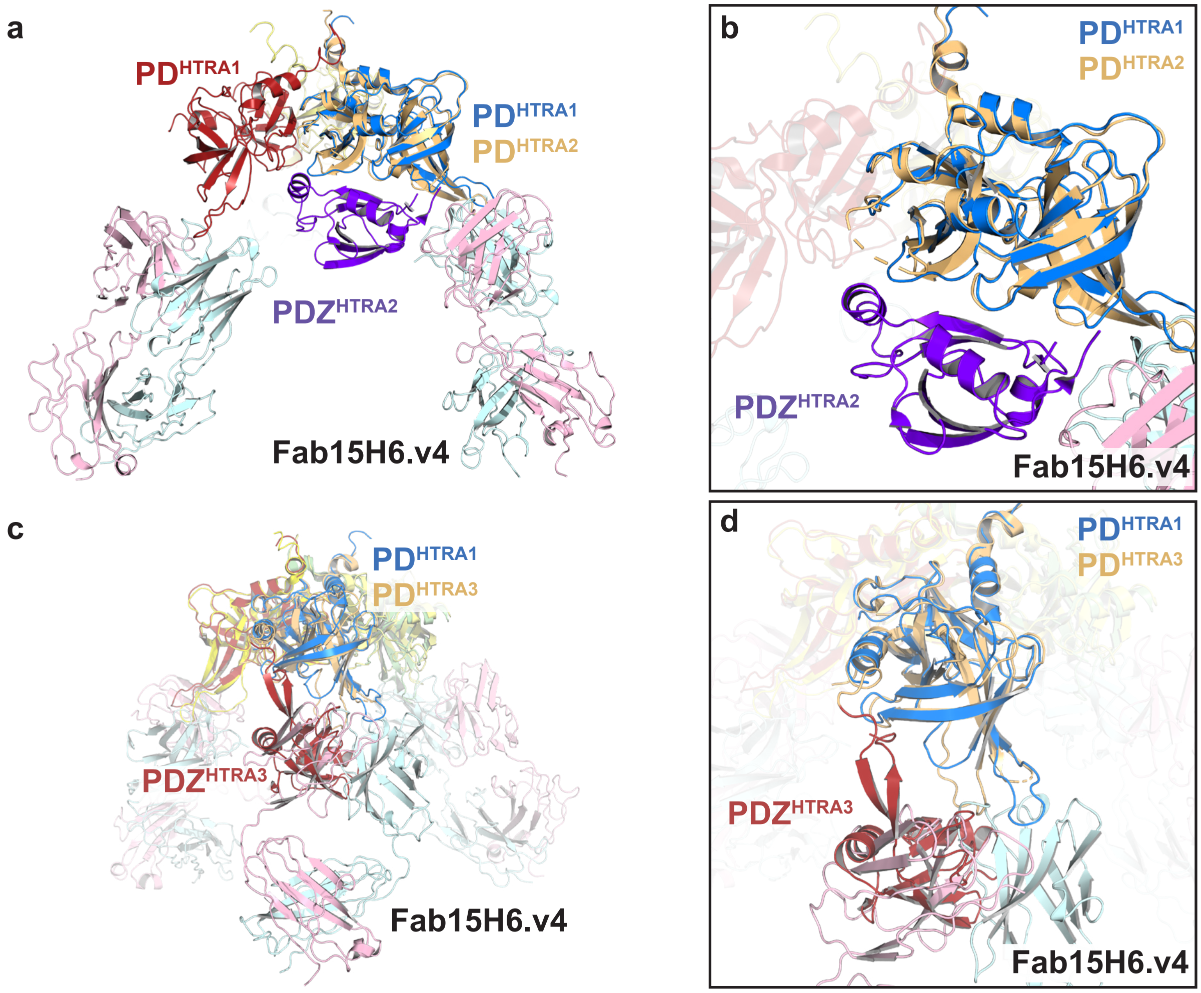

Supplementary Figure S10

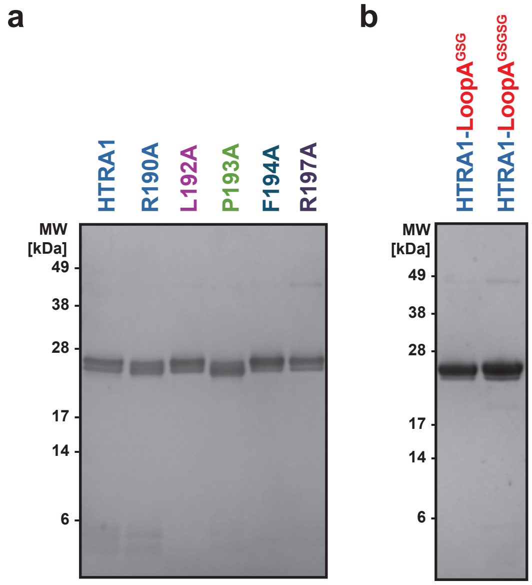

Supplementary Figure S11

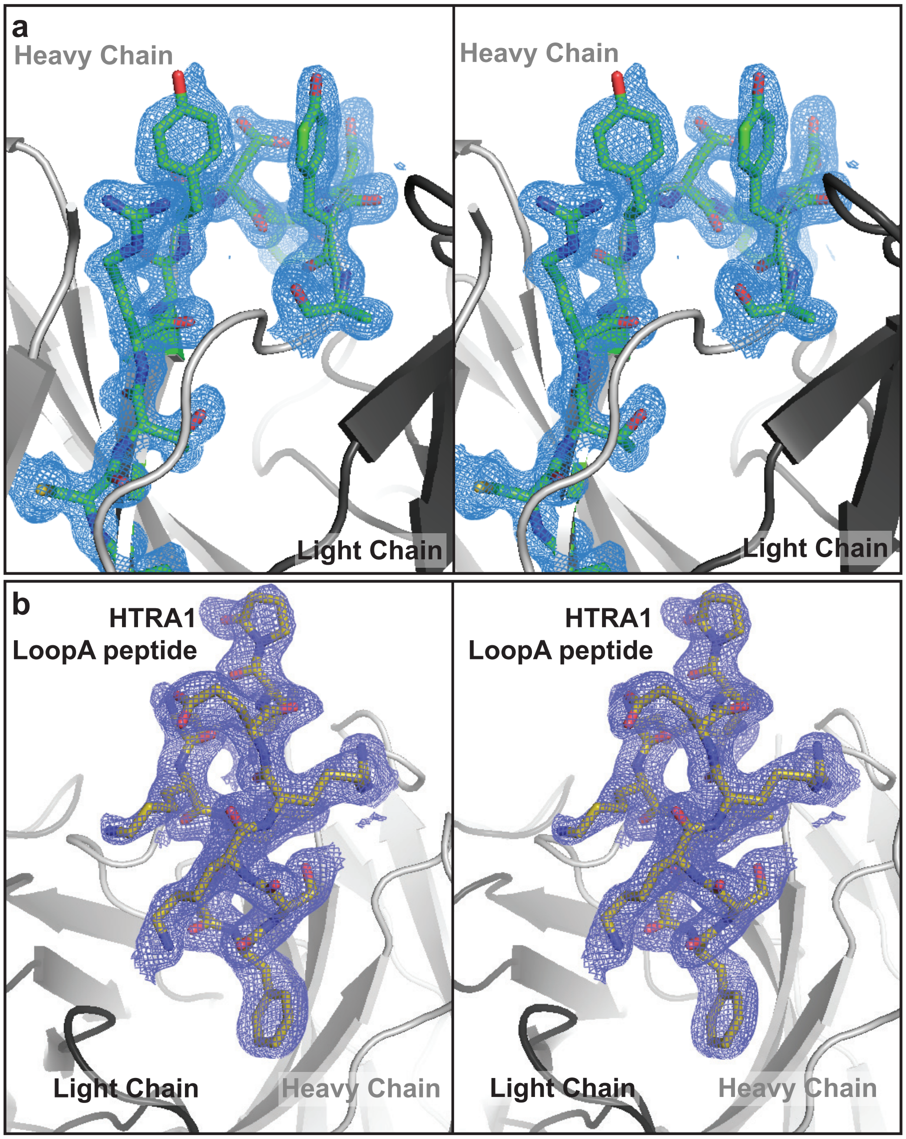

Supplementary Figure S12

Supplementary Figure Legends

Supplementary Figure 1: Kinetics of HTRA1 protease activity, binding kinetics and inhibition of HTRA1 by single chain Fv15H6.v4

a. Time-dependent cleavage of 4 µM DKK3 by 1 µM HTRA1^{FL} at 37 ^{o}C. **b.** Cleavage of 4 µM DKK3 (16 h/37 ^{o}C) with increasing concentrations of HTRA1^{FL}. **c.** and **d.** Cleavage of 4 µM BIGLYCAN (260 min/37 ^{o}C, **c.**) and 4 µM DECORIN (16 h/37 ^{o}C, **d.**) with increasing concentrations of HTRA1^{FL}. For **a.-d.** Reduced samples were loaded onto 12% NuPAGE Gel (Thermo Fisher Scientific), run in in 1x MES SDS Running buffer (Thermo Fisher Scientific) and stained with SimplyBlue SafeStain. Concentrations of HTRA1^{FL} to cleave 50% of substrates were quantified by gel scanning (Bio - Rad Gel Doc™ EZ Imager and quantification by ImageJ) and were 0.01-0.1 µM for DKK3 (16 hr incubation), 0.1-0.5 µM for DECORIN (16 hr incubation) and 0.01-0.1 µM for BIGLYCAN (260 min incubation). **e.** Titration of 10 nM HTRA1^{PD} trimer with increasing concentrations of Fab15H6.v4 (32 nM to 6.5 nM). Full inhibition is achieved at a concentration of 32.5 nM of Fab15H6.v4, indicating a 1:3 complex (HTRA1 trimer:Fab). **f.** Binding kinetics of HTRA1^{PD} and single chain Fv fragment of Fab15H6.v4 (scFv15H6.v4) determined by SPR. **g.** Enzymatic activity of HTRA1^{FL}, HTRA1^{PD} and HTRA1$^{PD/SA}$ in the presence of scFv15H6.v4. **h.** Labeling of HTRA1^{PD} using fluorescent TAMRA-ABP in the presence of scFv15H6.v4. Images in **a.** - **d.** and **h.** are representative of at least two independent experiments. Data in **e.**, **f.** and **g.** are presented as the mean ± S.D. of three independent experiments. Source data are provided as a Source Data file.

Supplementary Figure 2: Purification of HTRA1$^{PD/SA}$:Fab15H6.v4 complex and cryo-EM processing workflow

a. Peak fractions of the HTRA1$^{PD/SA}$:Fab15H6.v4 complex from Figure 1d were reduced and loaded onto 12% NuPAGE Gel (Thermo Fisher Scientific), run in in 1x MES SDS Running buffer (Thermo Fisher Scientific) and stained with SimplyBlue SafeStain. The HTRA1$^{PD/SA}$ and Fab co-migrate under reducing conditions (~25kDa). **b.** Representative micrograph of HTRA1$^{PD/SA}$:Fab15H6.v4 complex. **c.** 2D class averages of HTRA1$^{PD/SA}$:Fab15H6.v4 complex. **d.** Cryo-EM processing workflow using cisTEM. The obtained five 3D classes showed different angles of Fab15H6.v4 relative to the HTRA1 trimer. Visual inspection of each map showed similar conformational distortion in the active site. Further refined by RELION particle polishing using the best of the 5 maps enabled resolving cryo-EM maps with increased resolution. **e.** Isosurface rendering of HTRA1$^{PD/SA}$:Fab15H6.v4 maps with surface coloring according to local resolution. **f.** Detailed view on the catalytic center with local resolution estimates. **g.** Heatmaps representation on the distribution of assigned particle orientation generated by cisTEM. **h.** FSC curves for corrected reconstruction of HTRA1$^{PD/SA}$:Fab15H6.v4 maps. **i.** Density and atomic model for the catalytic center and LoopA of HTRA1$^{PD/SA}$ as well as the paratope of Fab15H6.v4. Image in **a.** is representative of at least two independent experiments. Source data are provided as a Source Data file.

Supplementary Figure 3: Purification of wildtype HTRA1^{PD}:Fab15H6.v4 complex and cryo-EM processing workflow

a. SEC elution profile of HTRA1^{PD}:Fab15H6.v4 complex (blue) on Sepharose 6 3.2/300 column (GE Healthcare) in comparison to molecular weight gel filtration standard (grey, Bio-Rad Laboratories). Complex peak fractions were reduced and loaded onto 12% NuPAGE Gel (Thermo Fisher Scientific), run in in 1x MES SDS Running buffer (Thermo Fisher Scientific) and stained with SimplyBlue SafeStain. The HTRA1^{PD} and Fab co-migrate under reducing conditions (~25kDa). **b.** Representative micrograph and **c.** 2D class averages of HTRA1^{PD}:Fab15H6.v4 complex. **d.** Data collection and processing scheme to obtain high resolution reconstructions of HTRA1^{PD}:Fab15H6.v4 complex. **e.** Cryo-EM map of HTRA1^{PD}:Fab15H6.v4 used for model building and refinement showing local resolution estimates. **f.** Detailed view on the catalytic center with local resolution estimates. **g.** Heatmap representation of the distribution of assigned particle orientation generated by cryoSPARC and FSC curves for corrected reconstruction of HTRA1^{PD}:Fab15H6.v4 maps. **h.** Map details showing active center loops and catalytic triad (H220, D250, S328) of HTRA1^{PD} as sticks combined with surface representation of the cryo-EM map of HTRA1^{PD}:Fab15H6.v4. **i.** Structural model of HTRA1^{PD}:Fab15H6.v4 shown as putty representation of the assigned b-factors, ramp-colored from blue to red representing low to high temperature factors. Detailed view on the HTRA1^{PD} catalytic center loops, showing significantly reduced b-factors. Cartoon representation in the background as guide. **j.** Density and atomic model for the catalytic center and LoopA of HTRA1^{PD} as well as the paratope of Fab15H6.v4. Chromatogram and image in **a.** as well as image in **b.** are representative of at least two independent experiments. Source data are provided as a Source Data file.

Supplementary Figure 4: Comparison of cryo-EM wild-type and mutant structures; surface electrostatics of HTRA1^{PD} and Fab15H6.v4 and comparison of LoopA conformations

a. Comparison of structures of HTRA1^{PD} (blue) and HTRA1$^{PD/SA}$ (pink) determined by cryo-EM **b.** Surface electrostatic potential of Fab15H6.v4 with bound LoopA peptide (yellow) showing negatively charged paratope (X-ray structure) **c.** HTRA1^{PD} monomer (cryo-EM) showing positively charged epitope of LoopA. Fab15H6.v4 is shown as cartoon representation. **d.** Fab-bound LoopA is twisted by > 20° and the ß-strand partially unfolds when compared to unbound, apo-HTRA1 crystal structures. Cryo-EM structures of Fab-bound HTRA1^{PD} and HTRA$^{PD/SA}$ (in blue and pink, respectively) compared to available HTRA1 crystal structures in wheat and green (pdbs 3TJN, 3NWU, 3NZI, 3TJO, 3NUM all feature similar conformations). V201, V199 and E198 indicate the boundary of LoopA and the ß-strand stem in Fab-bound HTRA1$^{PD/SA}$, Fab-bound HTRA1^{PD} and apo-HTRA1, respectively.

Supplementary Figure 5: Structural rearrangements of Fab-bound HTRA1$^{PD/SA}$ in comparison to available crystal structures

a. Structure of Fab-bound HTRA1$^{PD/SA}$ (pink) compared to competent HTRA1 in the active conformation (wheat, pdb 3TJN_chainB). The distorted LoopD, Loop1 and Loop2 of Fab-bound HTRA1 are indicated in salmon. **b.** Detailed view of structural rearrangements within catalytic center of HTRA1$^{PD/SA}$ shows the same distortions as observed in the Fab-bound wildtype HTRA1^{PD} (Figure 2e, f), including the H220 side chain flip, obstruction of S1 pocket access by L345 and a non-functional oxyanion hole. **c.** Comparison of Fab-bound HTRA1$^{PD/SA}$ (pink) and non-competent HTRA1^{PD} (green, pdb 3TJN_chainA) with LoopD, Loop1 and Loop2 in similar non-competent conformations (cryo-EM red, X-ray structure pdb 3TJN salmon).

Supplementary Figure 6: Molecular dynamics simulations of competent HTRA1^{PD} (pdb 3NZI) and PCA analysis

a. Measured distances between L345$^{C\gamma1}$ and S328$^{O\gamma}$ in the Molecular Dynamics (MD) simulations of monomeric competent HTRA1^{PD} (pdb 3NZI_chainA with removed covalent inhibitor). The calculated distances of competent and non-competent conformations are plotted by green and red dotted lines, respectively. **b.** Measured distances between H220$^{N\varepsilon2}$ and S328$^{O\gamma}$ in the MD simulations of monomeric competent HTRA1^{PD} (pdb 3NZI_chainA with removed covalent inhibitor). **c.** and **d.** Principal component analysis of MD simulations of HTRA1 (pdb 3NZI_chainA with removed covalent inhibitor and pdb 3TJN_chainB) and of Fab-bound HTRA1^{PD} (cryo-EM; "Fab trial") showing cross plots of the first three individual principal components. **e.** Eigenvalue spectrum of the MD simulations. **f.** Average RMSF plot of individual residues plotted along the HTRA1^{PD} sequence during MD simulation for Fab-bound HTRA1 (cryo-EM, blue), HTRA1-pdb 3NZI_chainA with removed covalent inhibitor (green) and HTRA1-pdb 3TJN_chainB (wheat).

Supplementary Figure 7: Comparison of Fab15H6.v4 structures, conservation map of HTRA1^{PD}, and superposition of HTRA2 and HTRA3 with the cryo-EM structure of the HTRA1^{PD}:Fab complex

a. Comparison of the crystal structures of apo-Fab15H6.v4 with LoopA peptide-bound Fab15H6.v4. **b.** Only slight structural rearrangements are observed in heavy chain CDR2 upon LoopA peptide binding. **c-d.** Conservation map rendering of HTRA1^{PD} (pdb 3NZI with covalent inhibitor removed), generated by using ConSurf Server [80] shows a highly conserved catalytic center and a variable LoopA region. The map is based on the alignment of HTRA proteins from 150 different species, including bacteria. **e.** Structural superpositions of HTRA2^{PD} (pdb 1LCY) and HTRA3^{PD} (pdb 4RI0) with the HTRA1^{PD}:Fab15H6.v4 complex to assess potential steric clashes of HTRA2^{PD}-loopA and HTRA3^{PD}-loopA with Fab15H6.v4 (light chain and heavy chain in pink and light blue, respectively). Indicated are the altered key residues within LoopA of HTRA2/3 (H170, P171 and L173 in HTRA2 and F166 in HTRA3 in salmon) and the residues of the LoopB of HTRA3 (N196-Q203 in red) that would cause steric clashes with the Fab15H6.v4.

Supplementary Figure 8: Conservation of HTRA1-LoopA in model organisms and inhibition of mouse HTRA1 catalytic activity by Fab15H6.v4; SEC elution profile for HTRA3-ΔLoopA mutant

a. Conservation of HTRA1 protease domain within vertebrate model organisms. LoopA sequences are completely conserved, except for zebrafish. **b.** Enzymatic activity and inhibition of the mouse HTRA1 protease domain (HTRA1^{PD}, orange) by Fab15H6.v4 compared to human HTRA1^{PD} (blue); **c.** Inhibition by the Fab15H6.v4 of mouse HTRA1^{PD} active site labeling using the fluorescent TAMRA-ABP assay. **d.** SEC elution profiles of wildtype HTRA3$^{PD/PDZ}$ (red) and HTRA3$^{PD/PDZ}$-ΔLoopA (purple) (molecular weight gel filtration standards in grey). **e.** No DKK3 cleavage was detected by the HTRA1^{PD}-LoopA^{HTRA3} or HTRA1^{PD}-LoopA^{HTRA4} chimeric proteins. Asterisks (*) indicate contaminant of the control Fab33 preparation. Right panel: cleavage assay control with individual proteins. Bar graphs in **b.** are presented as the mean ± S.D. of three independent experiments. Images shown in **c.** and **e.** as well as chromatogram in **d.** are representative of at least two independent experiments. Source data are provided as a Source Data file.

Supplementary Figure 9: Importance of LoopA for the catalytic activity of full-length HTRA1 (HTRA1^{FL}) and for binding of Fab15H6.v4

a. Enzymatic activity of HTRA1^{FL} compared to ΔLoopA mutant and chimeric HTRA1^{FL}-LoopA^{HTRA2} mutant. **b.** *In-vitro* cleavage of DKK3 by HTRA1^{FL} and LoopA mutants in the presence and absence of Fab15H6.v4. The Fab15H6.v4 inhibits DKK3 cleavage by HTRA1^{FL}, but not by the Loop A mutants. **c.** SPR binding kinetics for binding of Fab15H6.v4 to HTRA1^{FL}, to the ΔLoopA mutant and to chimeric HTRA1^{FL}-LoopA^{HTRA2}. **d.** The cryo-EM structure of HTRA1^{PD} with bound Fab15H6.v4 is superimposed on the structural model of full-length HTRA1 (HTRA1^{FL}) obtained by SAXS analysis [23]. The highlighted HTRA1 protomer (prot1) adopts an elongated conformation and the N-terminal IGFBP/Kazal-like domain and the C-terminal PDZ domain are connected to the central protease domain (PD) through flexible linkers. The other protomers 2 and 3 are indicated with a transparent outline. **e.** Close-up view shows that there is no steric conflict of the bound Fabs (shown are the light blue Fab heavy chain bound to protomer 2 and pink Fab light chain bound to protomer 3; Fab bound to protomer 1 is hidden behind protomer 1 and not visible in figure) with the IGFBP/Kazal-like domain (purple and red from protomer 1), which are positioned far away from the Fab epitope and do not interfere with Fab15H6.v4 binding to the HTRA1 trimer (protease domain of protomer 2 and 3 in yellow and dark red, respectively). **f.** Similarly, the C-terminal PDZ domain and Fab15H6.v4 do not overlap in our SAXS based model. Bar graphs in **a.** and binding kinetic data in **c.** are presented as the mean ± S.D. of three independent experiments. Images in **b.** are representative of at least two independent experiments. Source data are provided as a Source Data file.

Supplementary Figure 10: Overlay of HTRA2 and HTRA3 structures with Fab-bound HTRA1 structure

a. Overlay of HTRA2$^{PD/PDZ}$ (pdb 1LCY, wheat protease domain and purple PDZ) and Fab-bound HTRA1^{PD}:Fab15H6.v4 (protease domains of two protomers in blue and red; Fab in pink and light blue) structures. **b.** Detailed view on the PDZ domain of HTRA2. **c.** Overlay of HTRA3$^{PD/PDZ}$ (pdb 4RI0, wheat) with Fab-bound HTRA1^{PD}:Fab15H6.v4 structure (colors as in **a.**). **d.** Detailed view of the PDZ domain of HTRA3 (red) clashing with the light chain of Fab15H6.v4 (pink), when HTRA3 is overlayed with the HTRA1^{PD}:Fab15H6.v4 structure.

Supplementary Figure 11: Control gels showing individual HTRA1^{PD} proteins used for enzymatic assays. a. Wildtype HTRA1^{PD} and HTRA1^{PD}-LoopA mutants. **b.** HTRA1^{PD}-LoopA extension mutants used in Figure 5f for enzymatic assays. Images in **a.** and **b.** are representative of at least two independent experiments. Source data are provided as a Source Data file.

Supplementary Figure 12: Omit density maps for crystal structures of apo-Fab15H6.v4 and LoopA peptide-bound Fab15H6.v4

a. 2mFo-DFc omit maps of residues light chain 95-105 (in stereo) contoured at 1.0 σ for the apo-Fab15H6.v4 structure showing heavy chain in light grey and light chain in dark grey. Residues 95-105 are shown as sticks in green. **b.** 2mFo-DFc omit maps of LoopA peptide contoured at 1.0 σ for LoopA peptide:Fab15H6.v4 complex showing heavy chain in light grey and light chain in dark grey. Residues 190-201 of HTRA1 LoopA peptide are shown as sticks in light brown.

Supplementary Table 1: Binding kinetics of HTRA1 pre-incubated with ABP to Fab15H6.v4 and scFv15H6.v4 fragment

$HTRA1^{PD}$ + Analyte	k_a [M^{-1} s^{-1}] x 10^5	k_d [s^{-1}] x 10^{-5}	K_D [pM]
**$HTRA1^{PD}$ + Fab15H6.v4**	6.40 ± 0.28	7.55 ± 0.22	**118 ± 7.81**
**$HTRA1^{PD}$ + 7-mer ABP + Fab15H6.v4**	7.29 ± 0.56	15.0 ± 0.66	**207 ± 8.00**
**$HTRA1^{PD}$ + scFv15H6.v4**	41.9 ± 0.43	39.4 ± 3.04	**9420 ± 226**

Values indicated in the table are the mean ± S.D. of at least three independent experiments. Source data are available as a Source Data file.

Supplementary Table 2: Cryo-EM data collection, refinement, and validation statistics

	**HTRA1$^{PD/SA}$:Fab15H6.v4 EMD-25163, pdb 7SJO**		**HTRA1^{PD}:Fab15H6.v4 EMD-25162, pdb 7SJN**	
**Data collection and processing**				
Magnification	165.000 x	165.000 x	105.000 x	105.000 x
Voltage (kV)	300 kV	300 kV	300kV	300kV
Electron exposure (e–/Å^2)	65 e^-/Å	65 e^-/Å	64 e^-/Å	64 e^-/Å
Defocus range (μm)	0.5 – 1.5 μm	0.5 – 1.5 μm	0.5 – 1.5 μm	0.5 – 1.5 μm
Pixel size (Å)	0.416	0.416	0.419	0.419
Symmetry imposed	C1	C3	C3	C1
Initial particle images (no.)	505,669	505,669	566,088	566,088
Final particle images (no.)	219,676	219,676	184,782	184,782
Map resolution (Å)	3.3 Å	3.6 Å	3.3 Å	3.6 Å
FSC threshold	0.143	0.143	0.143	0.143
Map resolution range (Å)	9.04 - 3.3 Å	10.0 - 3.6 Å	8.97 – 3.3 Å	14.7 – 3.6 Å
**Refinement**				
Initial model used (pdb code)	HTRA1$^{PD/SA}$ (3TJO) Fab15H6.v4 (7SJM)	N/A	HTRA1$^{PD/SA}$ (3TJO) Fab15H6.v4 (7SJM)	N/A
Model resolution(Å)	10.0 - 3.6 Å (C3)<br>9.04 - 3.3 Å (C1)		8.97 – 3.4 Å (C3)<br>14.7 – 3.6 Å (C1)	
FSC threshold	0.143		0.143	
**Model composition**				
Non-hydrogen atoms				
Protein residues	14271		19062	
Ligands	1881		1251	
	0		0	
B factors (Å^2)				
Protein	69.53		89.45	
R.m.s. deviations				
Bond lengths (Å)	0.003 (0)		0.002 (0)	
Bond angles (°)	0.693 (12)		0.521 (0)	
**Validation**				
MolProbity score	1.75		1.56	
Clashscore	8.73		10.00	
**Ramachandran plot**				
Favored (%)	95.91		96.90	
Allowed (%)	4.09		3.10	
Disallowed (%)	0		0	

Supplementary Table 3: X-ray data collection and refinement statistics of apo-Fab15H6.v4 and Fab15H6.v4 + LoopA peptide

	**apo-Fab15H6.v4**<br>**pdb 7SJM**	**Fab15H6.v4 + LoopA peptide**<br>**pdb 7SJP**
**Data collection**		
Space group	C2	$P2_12_12_1$
Cell dimensions		
a, *b*, *c* (Å)	154.12, 60.34, 53.44	60.19, 90.21, 106.31
α, β, γ (°)	90.0, 97.32, 90.0	90.0, 90.0, 90.0
Resolution (Å)	39.47-1.8 (1.864-1.8)	36.45-2.1 (2.175-2.1)
R_{sym} or R_{merge}	0.083 (0.771)	0.107 (0.814)
$I / \sigma I$	11.2 (1.9)	12.7 (1.8)
Completeness (%)	97.27 (95.37)	99.6 (96.4)
Redundancy	3.5 (3.4)	6.4 (4.4)
CC ½	0.997 (0.608)	0.998 (0.675)
**Refinement**		
Resolution (Å)	1.8 Å	2.1 Å
No. reflections	153818 (8080)	221759 (11953)
R_{work} / R_{free}	0.1703 / 0.2015 (0.2528 / 0.2916)	0.1647 / 0.1988 (0.2429 / 0.2836)
No. atoms		
Protein	3326	3395
Ligand/ion	32	47
Water	469	410
B-factors		
Protein	25.00	37.20
Ligand/ion	64.00	92.80
Water	40.60	49.30
R.m.s. deviations		
Bond lengths (Å)	0.010	0.012
Bond angles (°)	1.08	1.79

One crystal was used for each structure *Values in parentheses are for highest-resolution shell.

Supplementary Table 4: Binding kinetics of Fab15H6.v4 to HTRA1^{PD} with mutated LoopA as determined by SPR

HTRA1^{PD} protein	k_a [M^{-1} s^{-1}] x 10^5	k_d [s^{-1}] x 10^{-3}	K_D [nM]
**R190A**	37.8 ± 6.74	16.1 ± 2.12	**3.47 ± 1.00**
**L192A**	5.15 ± 1.48	31.8 ± 10.8	**61.5 ± 4.70**
**P193A**	7.08 ± 1.97	27.7 ± 6.77	**40.2 ± 22.0**
**F194A**	22.7 ± 7.27	0.11 ± 0.09	**0.05 ± 0.02**
**R197A**	1.09 ± 0.27	13.6 ± 1.60	**133 ± 32.0**

Values indicated in the table are the mean ± S.D. of at least three independent experiments. K_D of wildtype HTRA1 to Fab15H6.v4 is 118 ± 7.81 pM (Supplementary Table 1). Source data are available as a Source Data file.

Supplementary Table 5: Binding kinetics of Fab15H6.v4 interaction with LoopA peptides as determined by SPR

LoopA peptide	k_a [M^{-1} s^{-1}] x 10^5	k_d [s^{-1}] x 10^{-3}	K_D [nM]
**HTRA1 LoopA peptide wildtype**	96.0 ± 35.2	7.42 ± 1.63	**0.77 ± 0.14**
**HTRA1 LoopA peptide R190A**	0.04 ± 0.01	4.30 ± 0.65	**1270 ± 416**
**HTRA1 LoopA peptide L192A**			**no binding**
**HTRA1 LoopA peptide P193A**			**no binding**
**HTRA1 LoopA peptide F194A**	49.7 ± 15.2	29.4 ± 6.36	**5.54 ± 0.44**
**HTRA1 LoopA peptide R197A**			**no binding**
**HTRA2 LoopA peptide wildtype**			**no binding**
**HTRA3 LoopA peptide wildtype**			**no binding**
**HTRA4 LoopA peptide wildtype**			**no binding**

Values indicated in the table are the mean ± S.D. of at least three independent experiments. Source data are available as a Source Data file.

Supplementary Table 6: Binding kinetics of wildtype HTRA1^{PD} interaction with Fab15H6.v4 mutants as determined by SPR

Fab15H6.v4 mutants	k_a [M^{-1} s^{-1}] x 10^5	k_d [s^{-1}] x 10^{-3}	K_D [nM]
**T30A (V_HCDR1)**	16.4 ± 1.35	3.59 ± 0.11	**2.19 ± 0.14**
**D31A (V_HCDR1)**	15.9 ± 2.38	4.15 ± 0.60	**2.65 ± 0.45**
**S32A (V_HCDR1)**	9.22 ± 0.71	1.78 ± 0.17	**1.82 ± 0.09**
**D52A (V_HCDR2)**	1.15 ± 0.04	30.0 ± 1.00	**260 ± 2.01**
**T91A (V_H)**	21.5 ± 5.10	3.14 ± 1.02	**1.19 ± 0.31**
**Y100A (V_HCDR3)**	0.89 ± 0.12	2.42 ± 0.74	**27.0 ± 1.02**
**D101A (V_HCDR3)**	10.0 ± 0.34	4.97 ± 0.24	**4.81 ± 0.20**
**Y102A (V_HCDR3)**	7.02 ± 0.32	4.95 ± 0.17	**7.45 ± 0.34**
**Combined (8xA)**			**no binding**

Values indicated in the table are the mean ± S.D. of at least three independent experiments. K_D of wildtype HTRA1 to Fab15H6.v4 is 118 ± 7.81 pM (Supplementary Table 1). Source data are available as a Source Data file.

Supplementary Table 7: Binding kinetics of Fab15H6.v4 to HTRA family and mutant proteins as determined by SPR

HTRA	k_a [M^{-1} s^{-1}] x 10^5	k_d [s^{-1}] x 10^{-3}	K_D
**HTRA1^{PD}-ΔLoopA**			**no binding**
**HTRA1^{PD}-LoopA^{HTRA2}**			**no binding**
**HTRA1^{PD}-LoopA^{HTRA3}**			**no binding**
**HTRA1^{PD}-LoopA^{HTRA4}**			**no binding**
**HTRA2 wildtype**			**no binding**
**HTRA2-LoopA^{HTRA1}**	33.0 ± 23.1	15.1 ± 1.54	**52.6 ± 15.9 pM**
**HTRA3 wildtype**			**no binding**
**HTRA3-LoopA^{HTRA1}**	63.2 ± 18.0	23.1 ± 3.85	**35.6 ± 13.8 pM**
**HTRA1^{FL}**	9.18 ± 2.43	45.2 ± 17.4	**0.54 ± 0.33 nM**
**HTRA1^{FL}-ΔLoopA**			**no binding**
**HTRA1^{FL}-LoopA^{HTRA2}**			**no binding**
